# Beta radiation for glaucoma surgery

**DOI:** 10.1590/S1516-31802012000300013

**Published:** 2012-07-12

**Authors:** James F. Kirwan, Christina Rennie, Jennifer R. Evans

## Abstract

**BACKGROUND:**

The outcome of glaucoma surgery can be affected by the rate at which the surgical wound heals. Beta radiation has been proposed as a rapid and simple treatment to slow down the healing response.

**OBJECTIVE:**

To assess the effectiveness of beta radiation during glaucoma surgery (trabeculectomy).

**CRITERIA FOR CONSIDERING STUDIES FOR THIS REVIEW:**

We searched the Cochrane Central Register of Controlled Trials (Central) in The Cochrane Library (which includes the Cochrane Eyes and Vision Group Trials Register) (Issue 4 2008), Medline (January 1966 to October 2008) and Embase (January 1980 to October 2008). The databases were last searched on 24 October 2008.

**SELECTION CRITERIA:**

We included randomized controlled trials comparing trabeculectomy with beta radiation to trabeculectomy without beta radiation.

**DATA COLLECTION AND ANALYSIS:**

We collected data on surgical failure (intraocular pressure > 21 mmHg), intraocular pressure and adverse effects of glaucoma surgery. We pooled data using a fixed-effect model.

**MAIN RESULTS:**

We found four trials that randomized 551 people to trabeculectomy with beta irradiation versus trabeculectomy alone. Two trials were in Caucasian people (126 people), one trial in black African people (320 people) and one trial in Chinese people (105 people). People who had trabeculectomy with beta irradiation had a lower risk of surgical failure compared to people who had trabeculectomy alone (pooled risk ratio (RR) 0.23 (95% CI 0.14 to 0.40). Beta irradiation was associated with an increased risk of cataract (RR 2.89, 95% CI 1.39 to 6.0).

**AUTHORS’ CONCLUSIONS:**

Trabeculectomy with beta irradiation has a lower risk of surgical failure compared to trabeculectomy alone. A trial of beta irradiation versus anti-metabolite is warranted.

This is the abstract of a Cochrane Review published in the Cochrane Database of Systematic Reviews (CDSR) 2009, Issue 2, Art. No. CD003433, DOI: 10.1002/14651858.CD003433.pub2 (http://onlinelibrary.wiley.com/doi/10.1002/14651858.CD003433.pub2/abstract). For full citation and authors details see reference 1.

For Latin America and the Caribbean, the full text is freely available from: http://cochrane.bvsalud.org/cochrane/show.php?db=reviews&mfn=1834&id=CD003433&lang=pt&dblang=&lib=COC&print=yes.

## References

[1] Kirwan JF, Rennie C, Evans JR (2009). Beta radiation for glaucoma surgery. Cochrane Database Syst Rev.

